# Antenatal care service satisfaction and associated factors among pregnant women at public health facilities of Wogera district, Northwest Ethiopia: a cross-sectional study

**DOI:** 10.3389/fgwh.2024.1422047

**Published:** 2024-08-01

**Authors:** Asrat Kassaw Belachew, Ayal Debie, Demiss Mulatu Geberu, Abinet Dagnew, Gedamnesh Bitew, Tadesse Mamo Dejene, Agmasie Damtew Walle

**Affiliations:** ^1^ Department of Public Health, School of Public Health, Asrat Woldeyes Health Science Campus, Debre Berhan University, Debre Birhan, Ethiopia; ^2^ Department of Health Systems and Policy, University of Gondar, Gondar, Ethiopia; ^3^ Department of Public Health, Wollo University, Dessie, Ethiopia; ^4^ Department of Health Informatics, School of Public Health, Asrat Woldeyes Health Science Campus, Debre Berhan University, Debre Birhan, Ethiopia

**Keywords:** antenatal care, pregnant women, satisfaction, northwest, Ethiopia

## Abstract

**Background:**

Pregnant women's satisfaction with Ante-Natal Care (ANC) is crucial for improving its quality and providing standardized healthcare services. However, studies on pregnant women's satisfaction with antenatal care and associated factors are limited in Ethiopia, particularly in the study area. Therefore, this study aimed to assess satisfaction and associated factors among pregnant women receiving antenatal care in Wogera district.

**Methods:**

A facility-based cross-sectional study was conducted in Wogera district from March to April 2024. A total of 458 pregnant women who attended ANC at health facilities were included in the study. Interviewer -administered structured questionnaire was used to obtain the necessary information for this study and systematic random sampling was used to select the study participants. Multivariable and binary logistic regression analysis was used to identify the effect of each independent variable on the outcome (satisfaction).

**Results:**

The overall satisfaction of antenatal care services among pregnant women was 92.1% (95% CI: 89.5, 94.5%). The majority (98.3%) of them were satisfied by the provider's greeting and 97.8% were satisfied by the cost of service but pregnant women were less satisfied by waiting time to see the health workers, cleanness of the toilet, and water supply. Satisfaction of pregnant women was associated with housewife occupational status [AOR = 3.05, 95% CI: 1.02, 9.15], civil servants occupational status [AOR = 4.02, 95% CI: 1.02, 15.85], age ≥25 [AOR = 2.78, 95% CI: 1.05, 1.74], advice on family planning [AOR = 7.29, 95% CI: 3.08, 17.05], one ANC visit [AOR = 3.61, 95% CI: 1.84, 8.74] and the respondents pregnant women who have ≥2 pregnancy [AOR = 4.55, 95% CI: 1.88, 11.03] were the predictors of level of satisfaction.

**Conclusion:**

Pregnant women's satisfaction with antenatal care was high, influenced by factors such as having two or more pregnancies, timing of the first ANC visit, family planning advice, age ≥25 years, and being a housewife or civil servant. Efforts should focus on reducing wait times, ensuring clean water access, and improving latrine hygiene at healthcare facilities to sustain this satisfaction. Specifically, facilities should streamline appointments, maintain safe drinking water sources, and upgrade toilets for better comfort and hygiene.

## Introduction

ANC is the care provided by skilled healthcare professionals for pregnant women to ensure the best health conditions for both the mother and baby during pregnancy (1).

Internationally, complications during pregnancy, childbirth, and the postnatal period have been the main causes of death and disability among reproductive-age women (2). Due to maternal causes, 10.7 million women in the world die before the age of 25 years. Accordingly, 2.7 million neonatal deaths and 2.6 million stillbirths were reported during 1990–2015 (3). Nearly, 99% of maternal deaths in the globe occur in low-income countries, especially in South Asia and Sub-Saharan Africa (2, 4). According to the 2016 Ethiopian Demographic Health Survey (EDHS), the maternal mortality rate in Ethiopia was 412 per 100,000 live births. If women had access to quality medical care during pregnancy, childbirth, and the postpartum period, maternal deaths could be removed (5).

Even though the estimation of service quality is complex, pregnant women satisfaction could be the indirect measurement of service quality. Patient satisfaction is a subjective and dynamic perception of the extent to which the patient's expected healthcare needs are met (6). Assessing to what extent patients are satisfied with health services is clinically relevant, as satisfied patients are more likely to comply with treatment, take an active role in their care, continue using medical services, and recommend centers to others (7). A satisfied pregnant woman promotes the utilization of the service to four to five people, while a dissatisfied pregnant woman on the other hand will complain 20 or more (8). It is also essential to identify the factors involved in dissatisfaction if a good health system is wanted.

Different studies conducted around the globe reported the satisfaction level with ANC service in Nepal is 38.4% (9). Women's satisfaction with ANC across developing countries differs from country to country. A study conducted in the Ondo State of Nigeria revealed that more than half of the women were satisfied with the service they received (10). Another study conducted in Pakistan reported that the general satisfaction of women was 70% on maternity services (11). Furthermore, one study in the Gambia pointed out that 79.9% of women were satisfied with the care they received at public health facilities (12).

Different studies have been conducted in Ethiopia and reported the satisfaction level with ANC service among pregnant women is found between 21.5% and 90%. For example, the study conducted in Jimma town revealed that 60.4%of the women were satisfied with ANC service (13). A similar study conducted in Sidama Zone revealed that 33% of the women were satisfied (14). The study conducted in the Demba district of Gamo Gofa Zone showed that pregnant women's satisfaction with the ANC services received was 21.5% (15). In the same way, the study conducted in Bahir-Dar special Zone has revealed that less than half 47.7% of the pregnant women were satisfied with the ANC service provided by eight public health facilities (16). According to the above studies, associated factors with satisfaction with ANC were the geographical location of health facility, types of health facilities, setting of facilities, amenities, absence of clean latrine, inadequate water supply, service area space and availability of drug and supply. There are also other socio-demographic, obstetric, technical, and interpersonal factors (like educational level, residence, perceived quality of care, knowledge on antenatal care, service provider skills, the privacy of care, getting information about the importance of breastfeeding, waiting time, interpersonal aspect of care, management aspects, type of pregnancy, history of stillbirth, time to start first ANC follow up, and parity) (13–17).

Even though pregnant women satisfaction is basic for further improvement of the quality of antenatal care and to provide standardized health care service for pregnant women, little is known about the level and factors associated with satisfaction in Ethiopia in general, and no data in the study area in particular. Therefore, this study aimed to assess the satisfaction of pregnant women towards ANC and associated factors in the Wogera district. Hence, the output of this study generates valuable information to fill the previous gap and is used for the improvement of ANC service delivered by the health system in the study area in particular and another similar setting.

## Methods

### Study design and setting

An institution-based cross-sectional study was conducted in Wogera district, Central Gondar Zone, Amhara National Regional State Northwest Ethiopia public Health facilities from March to April 2024. The district was found 778 km away from Addis Ababa (the capital city of Ethiopia) and 220 km from Bahir Dar (the capital city of the region). It has 8 Health centers, one primary hospital, 41 health posts, 4 primary private clinics, and 2 drug stores. The district has a total population of 220,566 of these 1,124,450 were males according to the 2007 Central Statistical Agency of Ethiopia (CSA) (18). The estimated number of pregnant mothers in the district was 8,080.

### Source and study population

All pregnant women attending ANC service at public health facilities in Wogera district, Northwest Ethiopia were the source population, whereas all pregnant women attending ANC service in the selected health institution of the district were the study population.

### Sample size determination and sampling procedure

The sample size was calculated using single population formula, an assumption of 95% Confidence Level (CL), the proportion of pregnant women who were satisfied with ANC service in Tigray was (83.9%) (19), 5% margin of error (w), 2 design effect and 10% non-response rate (16). Based on the above information the sample size was 458.

Initially, three health centers (Ambagiorgis HC, Gedebeye HC, and Tirgosige HC) were selected by using a simple random lottery method, and Wogera hospital was selected purposely because there is no other hospital in the district and in the Ethiopian context the service providers in hospital is a slightly different form health center. Then, the calculated sample was allocated proportionally based on the ANC attendants’ 1-month previous report of the health institution. Finally, the participants were selected by using the systematic random sampling technique with 3 interval.

### Variables and measurements

Satisfaction of pregnant women toward antenatal care services at public health facilities was a dependent variable. The independent variables were socio-demographic and economic factors (age, occupation, residence, monthly household income, maternal and paternal education status), obstetric history (numbers of pregnancies, numbers of ANC visits and initiation of ANC visits, abortion history, and stillbirth), organizational factors (availability of equipment, essential laboratory test, and drugs, waiting area, privacy room, electric city, clean latrine, and water), service provision related factors (History taking, physical examination, laboratory evaluation, provision of supply to the women).

**Pregnant women satisfaction with ANC** was measured by using twelve items of questions which is adapted from study on the quality of ANC service (20). The questionnaire contained five-point Likert scale items, with 1 and 5 indicating the lowest and highest levels of satisfaction, respectively. The level of satisfaction was measured by selecting a response ranging from 1 = strongly disagree, 2 = disagree, 3 = neutral, 4 = agree to 5 = strongly agree.

**Overall satisfaction level:** Pregnant women who scored 75% and above in the 12 satisfaction measuring items were categorized as satisfied and those who scored less than 75% as dissatisfied (21).

### Data collection instruments and procedures

A structured interviewer-administered questionnaire, chart review, and resource inventory checklists were prepared by reviewing a variety of studies (16, 17, 22–26). The questionnaire was prepared in English, translated into Amharic, and was back to English to check its consistency. The Cronbach's alpha coefficient for the variables was greater than 0.80. Four data collectors and two supervisors were recruited from outside selected facilities. The exit interview was conducted by trained BSC nurses on ANC clinics. Following interviews, further informed consent was requested from the healthcare provider to access medical records. Medical records of reviewed women were reviewed on the same day, to collect additional information on the care they received during their visit. Data on the availability of infrastructures in the health facilities were collected by conducting a resource inventory checklist. An inventory checklist was used to assess the service providers were provided without interruption.

### Data quality control

To ensure the data quality, one day training was given for 4 Bachelor of Science (BSc) nurse data collectors and 2 BSc nurse supervisors. A pre-test was conducted on 23 pregnant women (5% of the sample) at Tseda Health Center, and necessary corrections and modifications were made to the tool before the actual data collection. During data collection, supervisors checked the data for accuracy, consistency, and completeness.

### Data processing and analysis

The completed data were cleaned, coded, and entered into the Epi-Info version and exported to SPSS version 21 for analysis. Descriptive statistics, text narration, and tables were used to present the results. Binary logistic regression was performed for analysis. In the bi-variable logistic regression analysis *p*-value, less than 0.2 was used to select the candidate variables for multivariable logistic regression analysis. In the final multivariable logistic regression analysis model, a *p*-value less than 0.05 and an AOR with 95% CI were used to state associated factors.

### Ethical considerations

Before data collection, ethical approval was obtained from the Institutional Research Review Board of the Institute of Public Health, the University of Gondar, and the Department of Health System and Policy which approved the procedure. An official letter of Permission was obtained from the Wogera District Health Bureau. A letter of cooperation from the Wogera district health bureau was brought to the selected health facilities to get access to the study participants. The investigators were explained to the participants about the procedures, risks, and benefits of the study. Additionally, investigators also ensured that participants understood the information they provided to decide voluntarily whether they wanted to participate then written consent was obtained from each study participant. Finally, participant's privacy and confidentiality of the information were maintained through non-identifiers of the name.

## Results

### Socio-demographic and economic characteristics of participants

A total of 458 pregnant women answered the questionnaire with a response rate of 100%. Of the total study participants, 53.7% were ≥ in the 25-year age group, and the minimum and maximum ages of the participants were 17 and 47 years, respectively. More than half (57.9%) of the respondents were rural in residents. Regarding educational status, 14.6% of the participants were college and above. More than three-fourths of the respondents (76%) were housewives in their occupational status (Table 1).

**Table 1:** Socio-demographic and economic characteristics of pregnant women in Wogera district, Northwest Ethiopia, 2024 (*n* = 458).

Variable	Category	Frequency (%)	Percent (%)
Age in year	<24	212	46.3
	≥25	246	53.7
Residence	Urban	193	42.1
	Rural	265	57.9
Occupation	Housewife	348	76
	Civil servant	78	17
	Merchant	32	7
Maternal education	Unable to read and write	241	52.6
	Able to read and write	82	17.9
	Primary education	68	14.8
	Higher education	67	14.6
Monthly household income (ETB)	<1,000	59	12.9
	1,000–2,500	227	49.6
	2,500–5,000	114	24.9
	≥5,000	58	12.7

### Obstetric history

Out of the total participants, 69.7% were multigravida in the numbers of pregnancies, and 51.7% of them have had a repeated visit for ANC. Nearly 30% (28.4%) of participants started their ANC visits for their pregnancies in the third trimester of pregnancy and 5% of the participants had a history of abortion (Table 2).

**Table 2:** Previous and current pregnancy status of the pregnant women attending ANC follow-up clinic at Wogera district, Northwest Ethiopia, 2024 (*n* = 458).

Variable	Category	Frequency (*n*)	Percent (%)
Initiation of ANC visit	First trimester	27	5.9
	Second trimester	301	65.7
	Third trimester	130	28.4
Numbers ANC of visit	1st	221	48.3
	≥2 visit	237	51.7
History of abortion	Yes	78	17
	No	380	83
History of stillbirth	Yes	23	5
	No	435	95
Numbers of pregnancies	Primigravida	139	30.3
	Multigravida	319	69.7

### Organizational infrastructures and service provision factors

All of the health facilities had separate antenatal care counseling outpatient departments (OPD), whereas 91.5% of the facilities had maternity waiting areas for services. Latrines, skilled prenatal care providers, clinical management guidelines, examination coaches, BP apparatuses, fetoscope, weight scales, iron foliate, tetanus toxoid, treatment of syphilis, treatment of pregnancy-induced hypertension, deworming drugs, and HIV and HCG test kits were fully (100%) available during the data collection period. Out of the total data collection facilities, 86.5% had good access to electricity and clean water. Only half of the health facilities had hemoglobin test kits, while 86.0% had urine analysis, blood group, and stool test kits during data collection. Blood pressure, weight, fetal heartbeat, and fetal positions of babies were checked for 99.3%, 98.7%, 79.8%, and 70.3% of the pregnant women, respectively. Concerning laboratory investigations, 45.4%, 69.4%, and 93% had hemoglobin, blood group/RH, and HIV tests respectively. Accordingly, in prophylactic therapy, 96.1% of pregnant women received iron folate supplementation. Similarly, 35.5% and 68.0% of the women were informed about laboratory test results during antenatal care and place of delivery, respectively. In addition, 13.8%, 14.4%, 94.8%, and 84.7% of women had counseling about birth preparedness plans, proper nutrition, and family planning methods respectively (Table 3).

**Table 3:** Service provision-related factors among pregnant women attending ANC follow-up clinic at Wogera district, Northwest Ethiopia, 2024 (*n* = 458).

Antenatal service provision	Performed		Not performed	
	Frequency (*n*)	Percent (%)	Frequency (*n*)	Percent (%)
History taking				
Current obstetric history	457	99.8	1	0.2
Past obstetric history	457	99.8	1	0.2
Physical examination				
Weight	452	98.3	6	1.7
Height	2	0.4	456	99.6
Blood pressure	455	99.3	3	0.7
Eye conjunctiva/sclera	444	97.6	11	2.4
Position of the baby	322	70.3	236	29.7
Listen to fetal heartbeat	365	79.7	93	20.3
Edema	406	88.6	52	11.4
Laboratory evaluation				
Hemoglobin/hematocrit	208	45.4	250	54.6
RPR for syphilis	311	67.9	147	32.1
Blood group	318	69.4	140	30.6
RH factor	318	69.4	140	30.6
Urinalysis	314	67.5	151	32.5
Stool examination	96	21	362	79.
Preventive measure				
Iron folate supplementation	440	96.1	18	3.9
Mebendazoal provision	310	67.7	148	32.2
Tetanus toxoid	420	91.7	38	8.3

### Satisfaction of pregnant women toward antenatal care service

Overall, 92.1% of women were satisfied by the ANC services they received. The majority (98.3%) of them were satisfied by the provider's greeting, 97.8% were satisfied by the cost of service, and 96.9% were satisfied by the privacy during the consultation was maintained. However, pregnant women in terms of waiting time to see the health workers, information on the result of the test, and cleanness of the toilet and water supply were least satisfied (Table 4).

**Table 4:** Level of pregnant women satisfaction toward ANC service at public health facilities of Wogera district, Northwest Ethiopia, 2024 (*n* = 458).

Measurement items	Satisfied		Dissatisfied	
	(*n*)	%	(*n*)	%
The provider greeting was good in a friendly way	450	98.3	8	1.7
Waiting time was fair	353	77.1	105	22.9
The waiting area is adequate and with seat	367	80.1	91	19.9
The provider was easy to understood	135	70.5	323	29.5
The cost incurred for the service was fair	448	97.8	10	2.2
Privacy during consultation was maintained	444	96.9	14	3.1
The procedure was cleanliness and sanitation	421	91.9	37	8.1
The antenatal clinic has a clean latrine and adequate water supply	237	51.7	221	48.3
You feel that today received full information about ANC	424	92.6	34	7.4
You want to continue the rest of ANC visit to these health facilities	437	95.4	21	4.6
You recommend antenatal care for others	436	95.2	22	4.8
Are you happy to all service	435	95	23	5
Overall satisfaction	422	92.1	36	7.9

### Factors associated with satisfaction in antenatal care

Women's satisfaction with antenatal care was associated with women's occupation as a housewife, civil servant, age ≥25, advice on family planning, repeated ANC visits, and numbers of getting two or more pregnancies.

Pregnant women whose age group ≥25 were 2.78 times [AOR = 2.78, 95% CI: 1.05, 7.14] more likely satisfied than their counterparts. Similarly, women with housewife occupational status were 3.05 [AOR = 3.05, 95% CI: 1.02, 9.15] and civil servants were 4.02 times [AOR = 4.02, 95% CI: 1.02, 15.85] more likely satisfied than merchants**.**

Pregnant women who have two or more pregnancies were 4.55 times [AOR = 4.55, 95% CI: 1.88, 11.03] more likely to be satisfied than women who have one pregnancy. Regarding the frequency of antenatal care follow-up, pregnant women who visited the health facility one time were 3.61 times [AOR = 3.61, 95% CI: 1.84, 8.74] more likely to be satisfied compared to those visiting two and above for their ANC follow-up. Pregnant women who received advice on family planning were 7.29 times [AOR = 7.29, 95% CI: 3.08, 17.05] more likely to be satisfied than their counterparts (Table 5).

**Table 5:** Bi-variable and multivariable logistic regression analysis of factors associated with satisfaction of ANC service at public health facilities of Wogera district, Northwest Ethiopia, 2024 (*n* = 458).

Variables	Satisfaction level		COR (95%CI)	AOR(95%CI)	AOR (*p*-value)
	Satisfied	Dissatisfied			
Age (in a year)					
<24	184	28	1	1	
≥25	238	8	4.52 (2.02, 4.69)	2.78 (1.05, 7.41)	**0**.**04**
Occupation					
Housewife	323	25	2.98 (1.12, 7.92)	3.05 (1.02, 9.15	**0**.**046**
Civil servant	73	5	3.69 (0.95, 11.98)	4.02 (1.02, 15.85)	**0**.**047**
Merchant	26	6	1	1	
Numbers of pregnancy					
Prim gravid	116	23	1	1	
Multi gravid	306	13	4.67 (2.29, 9.52)	4.55 (1.88, 11.03)	**0**.**001**
Number of ANC visits					
First visit	211	10	2.60 (1.22, 5.22)	3.61 (1.84, 8.74)	**0**.**005**
≥2 visit	211	26	1	1	
History of abortion					
No	69	9	1	1	
Yes	353	27	1.71 (0.77, 3.79)	1.61 (0.66, 3.94)	0.296
History of stillbirth					
No	110	13	1	1	
Yes	312	23	1.60 (0.79, 3.27)	1.39 (0.63, 3.12)	0.142
Advice on family planning					
No	53	17	1	1	
Yes	369	19	6.22 (3.05, 12.73)	7.29 (3.08, 17.05)	**<0**.**0001**
Advice on breastfeeding					
No	38	6	1	1	
Yes	384	30	2.02 (0.79, 5.16)	1.42 (0.47, 4.27)	0.531
Listening of FHB					
No	89	4	1	1	
Yes	333	32	0.47 (0.16, 1.36)	0.51 (0.16,1.59)	0.246

Bold values indicates *P*-value <0.05.

## Discussion

This study identified the magnitude of satisfaction with ANC and associated factors among pregnant women at public health facilities in the Wogera district. Overall, 92.1% [95% CI: 89.5, 94.5%] of pregnant women were satisfied with the ANC services they received. This finding indicated that the majority of pregnant women were highly satisfied. The quality of antenatal care (ANC) services for pregnant women was high, as indicated by their satisfaction, which is an indirect measure of quality in health service organizations. When women are satisfied with ANC services, it means their expectations have been met. In this study satisfaction with ANC was in line with study conducted in Addis Ababa 90% (27) and Kazakhstani 90% (28) but it was higher than the studies conducted in Gamo Gofa 21.5% (15), Sidama Zone 33% (14), Bahr Dar 52.3% (16), Jimma 60.4% (13), West Guji Zone 66% (29), Arba Minch 68%, Tigray 83.9% (19), Sudan 38% (30), Nigeria 81.4%, Nepal 48.3% (9) and Eli-Beheira (58.9%) (31). On the other hand, our finding was lower than the study conducted in Tamil Nadu (98.6%) (32).

The possible reason for the variation might be due to differences in study time, socio-economic factors, and cultural differences between communities and countries. Almost all pregnant women received blood pressure (BP) monitoring and weight assessment. This finding was high but the fetal positioning 70.3% and FHB (79.7%) assessments were lower than the result of a study in Harari, Ethiopia (24). Furthermore, the maternal advice given on nutrition and places of delivery which covered 94.8% and 68.1% of the targets, respectively, in our work was higher than similar advices on nutrition and birth preparedness plan that covered 35.3% and 14.4%, respectively, of the participants reported by a study in Gondar (33). This variation might be due to the availability of basic equipment, infrastructure, workload, and the commitment of prenatal care service providers, resulting in inconsistencies in maternal healthcare information.

Pregnant women were highly satisfied with courtesy and respect (98.3%), the level of privacy during examination (96.9%). This finding was higher than the study conducted in Tigray which revealed 78.1% and 76.7% respectively (19). This variation may be due to the different behavior of the health worker and the availability of antenatal care outpatient departments. On the other hand, pregnant women were least satisfied by the waiting time to see the health workers, information on the result of the test, and cleanness of the toilet and water supply.

Accordingly, the factors that predict overall satisfaction towards antenatal care, women who have housewife and civil servant occupations were more satisfied than merchants. This finding was similar to that of the study conducted in the Gamo Gofa and West Guji zones (15, 29). The possible reason might be those pregnant women who were housewives get more adequate rest time for advice than the others. Additionally, pregnant women who were civil servants might have relatively enough time to get the service and to be consulted by the medical personnel. The respondents who were in the age category group greater than or equal to 25 were three times more likely satisfied than those 24 and less than the age group. The possible explanation could be that as the age of pregnant women increases, they become more aware and experienced regarding their ANC services. They perceive an improvement in service quality compared to previous ANC visits.

Additionally, with age, women often have greater experience in pregnancy and childbirth, which enhances their overall satisfaction with the care received. In terms of the number of pregnancies, pregnant women who have two or more pregnancies are more likely satisfied than pregnant women who have one pregnancy. The possible reason might be pregnant women who have one pregnancy might have a higher expectation than multigravida mothers about ANC because they haven't previous experience. However, they might not get compatible services according to their higher expectation. As a result, the satisfaction of pregnant mothers who have one pregnancy might be lower than those who have multigravida. Satisfaction with the ANC received was also influenced by the frequency of ANC visits.

Those respondents who visited the ANC clinic for one time were four times more likely to be satisfied than those who visited twice and more. The finding was in line with the study conducted in Gamo Gofa (15). This is because the WHO-focused antenatal care guidelines recommend that all pregnant women should receive comprehensive standard services during their first visit, as this is crucial for establishing effective ongoing care and increasing their overall satisfaction with the services they receive (34). High expectations of women about ANC service accomplished and healthcare providers take enough time to discuss pregnancy and pregnancy-related issues. Conversely, this finding is inconsistent with the study conducted in the West Guji zone, Arba Minch, and Riyadh (29, 35). A highly satisfactory visit allows a woman to voice her concerns, which can increase her positive feelings toward the services. Additionally, a high number of visits can strengthen the positive relationship between providers and pregnant women, leading to maximum satisfaction with antenatal care services. This probable explanation was also supported by Tanzanian finding (36). The study also showed that pregnant women who got advice on family planning were seven times more likely satisfied than women who were not advised. This might be explained by the fact that pregnant women who received advice on family planning gained better information about recommended methods for lactating mothers, the importance of family planning, and birth spacing. This comprehensive information likely contributed to their higher satisfaction with ANC services.

### Limitations of the study

This study did not include qualitative data, which would have provided additional support for its findings. Additionally, the results may be subject to social desirability bias because the interviews with mothers were conducted exclusively within health facilities. This setting might have influenced the mothers to give more favorable responses than they would have in a different environment.

## Conclusion

Pregnant women's satisfaction with antenatal care was high. Key factors contributing to this satisfaction included having two or more pregnancies, the timing of the first ANC visit, and receiving advice on family planning, being aged ≧25 year, being a housewife and civil servant. To maintain and improve this satisfaction, emphasis should be placed on reducing waiting times, ensuring a clean water supply, and improving the cleanliness and construction of latrines. Specifically, healthcare facilities should streamline appointment processes to minimize waiting times, regularly maintain and clean water sources to guarantee safe drinking water, and invest in better toilet facilities to ensure hygiene and comfort for pregnant women.

## Data Availability

The raw data supporting the conclusions of this article will be made available by the authors, without undue reservation.

## References

[1] World Health Organization. Global Health Observatory (GHO) Data. Switzerland: World Health Organization (2015).

[2] Ashraf F Thaver IH Imtiaz F Ayub A. Quality assessment of focused antenatal care service delivery in tertiary care health facility. J Ayub Med Coll Abbottabad. (2017) 29(2):219–24. PMID: 28718235

[3] Sexual, W.H.O.J. and r. health. New Guidelines on Antenatal Care for a Positive Pregnancy Experience. Geneva, Switzerland: World Health Organization (2016). 28079998

[4] Demographic, S.A. Health Survey 2016: Key Indicator Report. Statistics South Africa. Pretoria, South Africa: National Department of health (2017).

[5] World Health Organization. Trends in Maternal Mortality: 1990 to 2008. Estimates Developed by wHO, UNICEF, UNFPA and the World Bank. Geneva, Switzerland: World Health Organization (2010).

[6] World Health Organization. Pregnant Women Satisfaction Evaluation. Geneva, Switzerland: World Health Organization (2000).

[7] Pascoe GC. Patient satisfaction in primary health care: a literature review and analysis. Eval Program Plann. (1983) 6(3–4):185–210. 10.1016/0149-7189(83)90002-2 10299618

[8] Press I Ganey RF Malone MP. Satisfied patients can spell financial well-being. Healthc Financ Manag J Healthc Financ Manag Assoc. (1991) 45(2):34–6. 38, 40–2. PMID: 10145381

[9] Bastola P Yadav DK Gautam H. Quality of antenatal care services in selected health facilities of Kaski district, Nepal. Int J Community Med Public Health. (2018) 5(6):2182–9. 10.18203/2394-6040.ijcmph20182142

[10] Fatile OA Akpor OA Okanlawon FA Fatile EO. Women and Providers’ Perception, Attitude and Satisfaction with Focused Antenatal Care in Ondo State, Nigeria. Hooghly, West Bengal, India: SCIENCEDOMAIN (2016).

[11] Ashraf M Ashraf F Rahman A Khan R. Assessing women’s satisfaction level with maternity services: evidence from Pakistan. Int J Collab Res Intern Med Public Health. (2012) 4(11):1841–51.

[12] Jallow IK Chou Y-J Liu T-L Huang N. Women’s perception of antenatal care services in public and private clinics in the Gambia. Int J Qual Health Care. (2012) 24(6):95–600. 10.1093/intqhc/mzs033 22789667

[13] Chemir F Alemseged F Workneh D. Satisfaction with focused antenatal care service and associated factors among pregnant women attending focused antenatal care at health centers in Jimma town, Jimma zone, south west Ethiopia; a facility based cross-sectional study triangulated with qualitative study. BMC Res Notes. (2014) 7(1):164. 10.1186/1756-0500-7-164 24646407 PMC3994781

[14] Tesfaye D Mekonnen A Negesa B. Maternal antenatal care service satisfaction and factors associated with rural health centers, Bursa district, Sidama zone, Southern Ethiopia: a cross-sectional study. J Women’s Health Care. (2017) 6(363):2167. 10.4172/2167-0420.1000363

[15] Mekonnen N Berheto TM Ololo S Tafese F. Quality of antenatal care services in Demba Gofa Woreda, Gamo Gofa zone, rural Ethiopia. Health Sci J. (2017) 11(3):502. 10.21767/1791-809X.1000502

[16] Ejigu T Woldie M Kifle Y. Quality of antenatal care services at public health facilities of Bahir-Dar special zone, Northwest Ethiopia. BMC Health Serv Res. (2013) 13(1):443. 10.1186/1472-6963-13-443 24161007 PMC4231347

[17] Abebe S Alemayehu A Gebremariam A Dirar A. Quality of antenatal care service in public health facilities of Chencha district, Gamo Gofa zone, Southern Ethiopia. MOJ Womens Health. (2017) 4(3):00086. 10.15406/mojwh.2017.04.00086

[18] Authority, C.S. 2007 Population and Housing Census Of Ethiopia. IPUMS international (2012).

[19] Fseha B. Assessment of mothers level of satisfaction with antenatal care services provided at Alganesh health center Shire, north west Tigray, Ethiopia. Biomed J Sci Tech Res. (2019) 16(1):11798–802. 10.26717/BJSTR.2019.16.002803

[20] Ejigu T Woldie M Kifle Y. Quality of antenatal care services at public health facilities of Bahir-Dar special zone, Northwest Ethiopia. BMC Health Serv Res. (2013) 13(1):1–8. 10.1186/1472-6963-13-443 24161007 PMC4231347

[21] Tayelgn A Zegeye DT Kebede Y. Mothers’ satisfaction with referral hospital delivery service in Amhara region, Ethiopia. BMC Pregnancy Childbirth. (2011) 11(1):1–7. 10.1186/1471-2393-11-78 22023913 PMC3254067

[22] Donabedian A. The quality of care: how can it be assessed? JAMA. (1988) 260(12):1743–8. 10.1001/jama.1988.03410120089033 3045356

[23] Town fi. Assessment of Quality of ANC Service and its Association with Intention to Deliver in Public Health Facilities in Bishoftu Town, Oromia, Ethiopia. Ethiopia: Doctoral Dissertation, Addis Ababa University (2017).

[24] Teji D Tekelehaymanot G Mesfin D. Assessment of Quality of Antenatal Care Service Provision and Associated Factor at Governmental Health Facilities of Harar Town Eastern Ethopia. Brussels, Belgium: Haramaya University (2017).

[25] Tesfaye T. Assess Quality of Antenatal Care Services in Rural Health Centers in Bursa Woreda, Sidama Zone, Southern Nations Nationalities People’s Region, Ethiopia, 2014. Ngong Rd, Nairobi, Kenya: Doctoral dissertation, Addis Ababa University (2014).

[26] Heaman MI Sword WA Akhtar-Danesh N Bradford A Tough S Janssen PA Quality of prenatal care questionnaire: instrument development and testing. BMC Pregnancy Childbirth. (2014) 14(1):188. 10.1186/1471-2393-14-188 24894497 PMC4074335

[27] Muzemil A. Assessment of Quality of Antenatal Care in Selected Hospitals in Addis Ababa, 2014. Ngong Rd, Nairobi, Kenya: Addis Ababa University (2014).

[28] Dauletyarova MA Semenova YM Kaylubaeva G Manabaeva GK Toktabayeva B Zhelpakova MS Are Kazakhstani women satisfied with antenatal care? Implementing the WHO tool to assess the quality of antenatal services. Int J Environ Res Public Health. (2018) 15(2):325. 10.3390/ijerph15020325 29438330 PMC5858394

[29] Selgado MB Dukele YH Amamo DD. Determinants of focused antenatal care service satisfaction in public health facilities in Ethiopia 2018: a mixed study design. J Public Health Epidemiol. (2019) 11(8):158–69. 10.5897/JPHE2019.1154

[30] Za Z aM I Nm B. Satisfaction among pregnant women towards antenatal care in public and private care clinics in Khartoum. Khartoum Med J. (2012) 4(2):146–56. Corpus ID:

[31] Ismail NIAA Essa RM. Pregnant women’s satisfaction with the quality of antenatal care at maternal and child health centers in El-Beheira governorate. Therapy. (2017) 14:15. 10.9790/1959-0602093646

[32] Sugunadevi G. Quality of antenatal care services at subcentres: an infrastructure, process and outcome evaluation in a district in Tamil Nadu. Int J Community Med Public Health. (2017) 4(11):4071–7. 10.18203/2394-6040.ijcmph20174647

[33] Worku AG Yalew AW Afework MF. Availability and components of maternity services according to providers and users perspectives in north Gondar, Northwest Ethiopia. Reprod Health. (2013) 10(1):43. 10.1186/1742-4755-10-43 23968306 PMC3765091

[34] World Health Organization. WHO Recommendations on Antenatal Care for a Positive Pregnancy Experience. Geneva, Switzerland: World Health Organization (2016). 28079998

[35] Kamil A Khorshid E. Maternal perceptions of antenatal care provision at a tertiary level hospital, Riyadh. Oman Med J. (2013) 28(1):33. 10.5001/omj.2013.07 23386942 PMC3562985

[36] Gupta S Yamada G Mpembeni R Frumence G Callaghan-Koru JA Stevenson R Factors associated with four or more antenatal care visits and its decline among pregnant women in Tanzania between 1999 and 2010. PLoS One. (2014) 9(7):e101893. 10.1371/journal.pone.0101893 25036291 PMC4103803

